# Tuberculosis Presenting as Multiple Pulmonary Nodules Mimicking Malignancy

**DOI:** 10.1177/23247096261429205

**Published:** 2026-03-04

**Authors:** Abdel Martinez, Fatimah Bello, Brandon Cantazaro, Luisa Montoya, Frank Mendiola

**Affiliations:** 1 The University of Texas Rio Grande Valley School of Medicine, Weslaco, USA; 2 Internal Medicine, Knapp Medical Center, Weslaco, TX, USA

**Keywords:** infectious disease, pulmonary critical care, radiology/imaging, tuberculosis, multiple pulmonary nodules

## Abstract

Multiple pulmonary nodules often raise concern for metastatic malignancy; however, the differential diagnosis is broad and includes infectious, inflammatory, granulomatous, vascular, and benign etiologies. Tuberculosis (TB), although uncommon, can present with multiple nodules that closely mimic metastatic disease on advanced imaging, requiring careful clinicoradiologic and microbiologic correlation. We describe a woman in her 50s who presented with dyspnea and new-onset heart failure, in whom imaging revealed mediastinal lymphadenopathy and bilateral pulmonary nodules concerning for malignancy. Evaluation was limited by concurrent COVID-19 infection, and CT-guided biopsy demonstrated necrotizing granulomas without evidence of malignancy or infection. She later re-presented with fever and productive cough, with imaging showing progression to cavitary lung lesions. Bronchoscopy was deferred due to high procedural risk, and sputum studies ultimately confirmed pulmonary TB by positive AFB smear and MTB PCR. She was started on standard antituberculous therapy and discharged with directly observed treatment. This case highlights the importance of maintaining a broad differential diagnosis when evaluating multiple pulmonary nodules and emphasizes the role of microbiologic testing in establishing the diagnosis when invasive procedures are not feasible.

## Background

Multiple pulmonary nodules classically raise concern for metastatic malignancy, but the differential diagnosis is broad and includes infectious etiologies (such as tuberculosis [TB], nontuberculous mycobacteria, and fungal infections), inflammatory or granulomatous diseases, vascular or embolic processes, and various benign lesions.^1^ Although uncommon, TB can present with multiple discrete nodules that closely mimic metastatic disease on CT or PET imaging.^2^ Because of this overlap, careful clinicoradiologic assessment combined with targeted microbiologic studies—and when necessary, histologic evaluation—is essential to avoid misdiagnosis and to guide appropriate management.^1,3^

## Case

A female patient in her 50s with no known medical history. Social history was notable for residence in South Texas near the U.S.–Mexico border; she lived in Mexico during childhood. She denied prior homelessness, incarceration, known sick contacts, or history of latent TB infection. She is currently unemployed and lives with her daughter. She denied tobacco, alcohol, or illicit substance use and reported a middle-level education. She presented to the emergency department with 1 week of shortness of breath accompanied by bilateral lower-extremity edema, frontal headache, nonproductive cough, orthopnea, and paroxysmal nocturnal dyspnea. On admission, she was hypertensive and found to have hypokalemia (K 3.0). An evaluation for secondary hypertension was performed, but serum aldosterone and ACTH levels were within normal limits. Her HbA1c was 11.6%. BNP was elevated at 4900 pg/mL. Transthoracic echocardiography revealed an LVEF of 15% to 20%. She was treated with diuretics and initiated on guideline-directed medical therapy.

**Figure 1.**
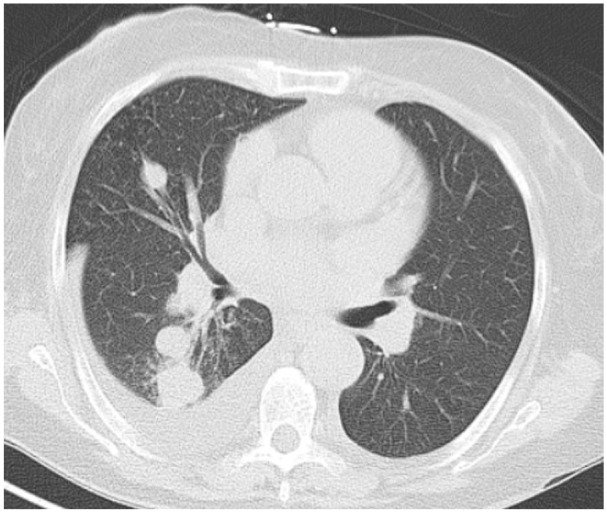
CT chest without contrast showed nodular lesions and a right-sided pleural effusion.

Chest radiography showed cardiomegaly with interstitial pulmonary edema and a pulmonary nodule. CT of the chest confirmed scattered mediastinal lymphadenopathy and nonspecific bilateral pulmonary nodules with minimal pleural effusions (Figure 1). Primary versus metastatic lung malignancy remained a concern. A CT of the abdomen and pelvis demonstrated partially calcified lesions over the left adrenal gland. Given her unintentional weight loss of more than 10 pounds, absence of night sweats, and mediastinal lymphadenopathy, there was a high suspicion for malignancy. Laboratory evaluation also showed a total protein level of 6.9 g/dL and hypoalbuminemia with an albumin level of 2.0 g/dL, suggesting poor nutritional status in the setting of chronic illness.

During this admission, the patient also tested positive for COVID-19 and was treated with remdesivir. Pulmonology was consulted for possible bronchoscopy; however, due to active COVID infection, interventional radiology—guided biopsy was recommended instead. CT-guided biopsy of a pulmonary nodule showed necrotizing granuloma, negative for malignancy, and negative for fungal and acid-fast bacilli staining. Tissue cultures were not obtained at that time. Unfortunately, the patient was lost to follow-up due to insurance constraints.

**Figure 2.**
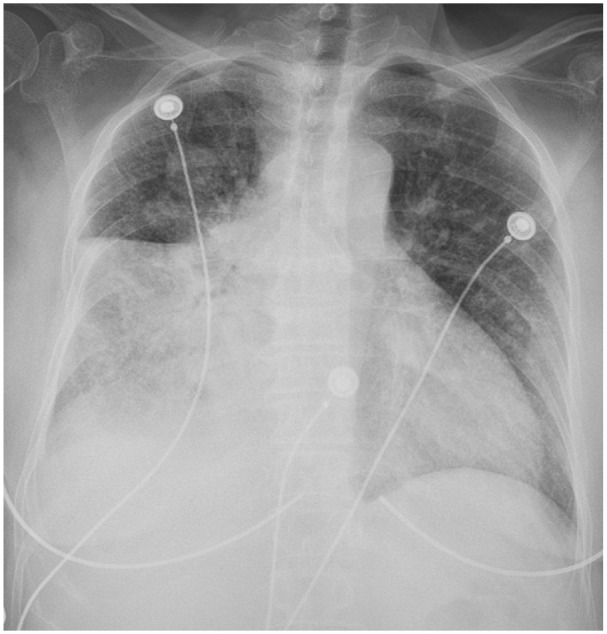
CXR showed a large right lung infiltrate involving the right middle lobe and right lower lobe.

**Figure 3.**
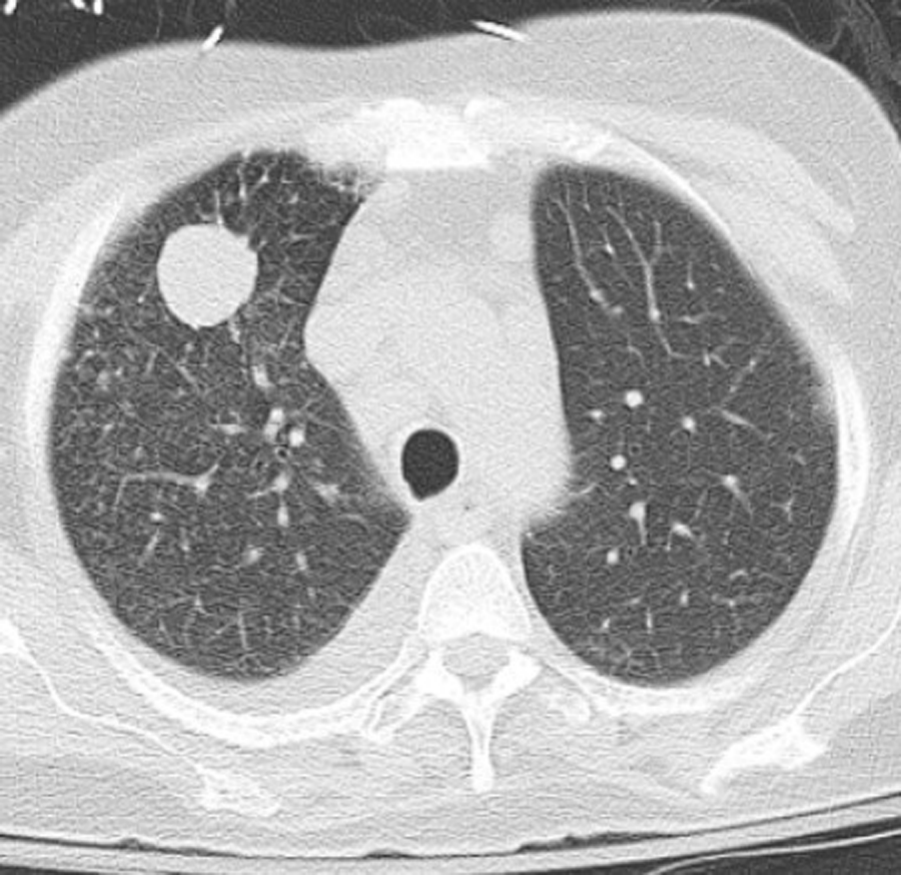
CT chest without contrast demonstrated a solitary cavitary pulmonary lesion in the right lung, associated with a small pleural effusion.

**Figure 4.**
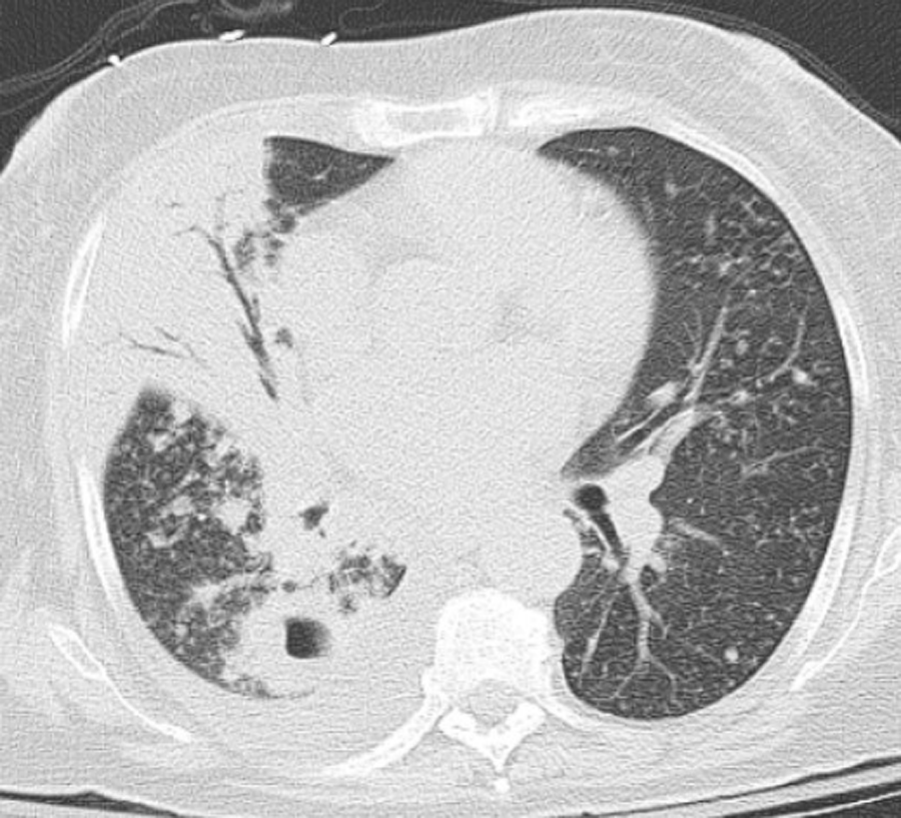
CT chest without contrast demonstrated a moderate-sized right lung infiltrate with multiple bilateral pulmonary nodules and pulmonary masses, some of which were cavitary.

**Figure 5.**
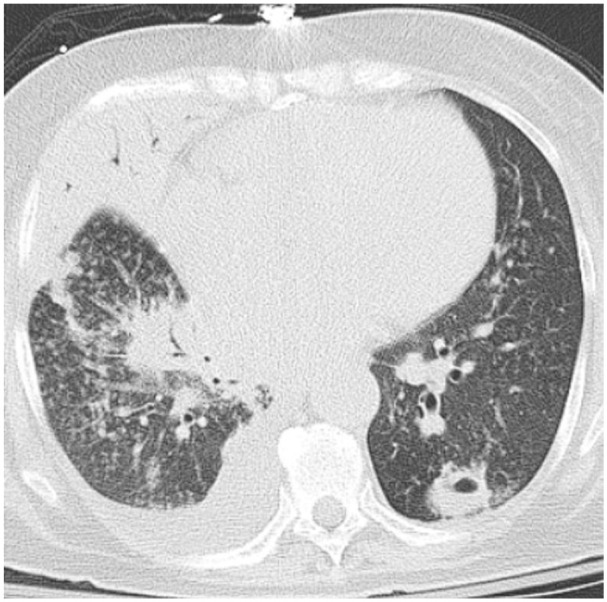
CT chest without contrast demonstrated right-sided pleural effusion with cavitary pulmonary lesions.

She was readmitted 8 months later with worsening shortness of breath, fever, and productive cough. Chest radiography revealed a large infiltrate involving the right middle and lower lobes (Figure 2). COVID, influenza, and a respiratory pathogen panel were negative. MRSA nasal screening was positive. Additional labs showed negative ANCA, elevated ACE (89 U/L), negative rheumatoid factor, ESR 58 mm/hour, elevated CRP, and negative HIV testing. Repeat CT chest demonstrated a moderately sized right-lung infiltrate and bilateral pulmonary nodules and masses, some of which were cavitary (Figures 3-5). Bedside point-of-care ultrasound revealed a small pleural effusion not amenable to thoracentesis.

Bronchoscopy was planned, but ultimately canceled after the patient declined the procedure due to perceived high risk. Subsequent AFB smear returned positive, and MTB PCR confirmed Mycobacterium TB infection. The patient was started on anti-tuberculous therapy with rifampin, isoniazid, pyrazinamide, and ethambutol. The county health department was notified, and she was deemed safe for discharge with directly observed therapy.

## Conclusion

This case highlights the importance of maintaining a broad differential diagnosis when evaluating multiple pulmonary nodules.^1^ Although metastatic malignancy is often the initial concern, infectious and inflammatory etiologies—including sarcoidosis, fungal infections, and mycobacterial diseases—must also be carefully considered.^1,4^ In this patient, the early suspicion for malignancy shifted when biopsy results demonstrated necrotizing granulomas negative for malignancy, and subsequent imaging showed progression from nodules to cavitary lesions, findings more consistent with granulomatous or infectious processes.^2,3^ The diagnostic evaluation was further complicated by limitations in performing bronchoscopy due to concurrent COVID-19 infection and severely reduced left ventricular ejection fraction.^5^ Ultimately, the presence of a productive cough, a positive AFB smear, and confirmatory MTB PCR established the diagnosis of pulmonary TB.^6^ This case underscores the need for a systematic diagnostic approach, the value of microbiologic testing when invasive procedures are not feasible, and the importance of considering a broad differential diagnosis when encountering a patient with multiple lung nodules.^1,6^
